# An integrative methodology for classifying physical activity level in apparently healthy populations for use in public health

**DOI:** 10.26633/RPSP.2017.161

**Published:** 2017-12-05

**Authors:** Carlos Cristi-Montero

**Affiliations:** 1 IRyS Group, Physical Education School Pontificia Universidad Católica de Valparaíso, Viña del Mar Chile IRyS Group, Physical Education School, Pontificia Universidad Católica de Valparaíso, Viña del Mar, Chile.

**Keywords:** Sedentary lifestyle, exercise, surveys and questionnaires, accelerometry, health, Estilo de vida sedentario, ejercicio, encuestas y cuestionarios, acelerometría, salud, Estilo de vida sedentário, exercício, inquéritos e questionários, acelerometria, saúde

## Abstract

Physical inactivity is one of the most important risk factors contributing to morbidity and mortality in the world, although sedentary behavior (SB), low-intensity physical activity (LIPA), and shorter sleep duration have also been associated with various chronic diseases and physiopathological conditions that may affect health, irrespective of one’s level of physical activity (PA). Current methods to evaluate and classify the PA level in the population appear to be limited, as they primarily focus on time spent performing moderate-to-vigorous PA (MVPA). The aim of this article is to analyze the scientific literature in regard to various combinations of patterns among sleep, SB, LIPA, and MVPA, in order to propose a more integrative PA classification in apparently healthy children, adolescents, and adults. In general, the most common classification is composed of four categories that combine MVPA with SB level as follows: i) “physically active” or “physically inactive” (meets or does not meet weekly MVPA recommendations) and ii) “high SB” or “low SB” (depending on amount of accumulated sedentary time per day).There is a consensus regarding the classification of physically active or not, but agreement has not been reached on the classification of a high SB or low SB level. This new, integrative approach appears to be an appropriate methodological proposal for categorizing the level of PA, with the aim of providing health professionals and researchers a more comprehensive vision of PA behaviors among the population.

## RESUMEN

La inactividad física es uno de los factores de riesgo más importantes que aumentan la morbilidad y mortalidad en el mundo, aunque el sedentarismo, la actividad física leve y una menor duración del sueño también se han asociado con diversas afecciones fisiopatológicas y enfermedades crónicas que pueden afectar la salud, independientemente del nivel de actividad física. Los métodos usados hoy en día para evaluar y clasificar el nivel de actividad física de la población parecen tener limitaciones, pues se centran principalmente en el tiempo dedicado a la actividad física de moderada a vigorosa. El objetivo de este artículo es analizar la bibliografía científica relacionada con diversas combinaciones de hábitos en lo referente al sueño, el sedentarismo, la actividad física leve y la actividad física de moderada a vigorosa, a fin de proponer una clasificación más integradora de la actividad física que realizan niños, adolescentes y adultos aparentemente sanos. En general, la clasificación más común consta de cuatro categorías que combinan la actividad física de moderada a vigorosa con el nivel de sedentarismo de la siguiente manera: 1) “físicamente activo” o “físicamente inactivo” (sigue o no las recomendaciones sobre cuánta actividad física de moderada a vigorosa debe realizarse cada semana); y 2) “nivel alto de sedentarismo” o “nivel bajo de sedentarismo”, dependiendo de la cantidad de tiempo dedicado a actividades sedentarias que se acumule por día. Hay consenso sobre la clasificación de físicamente activo o físicamente inactivo, pero no se ha llegado a un acuerdo con respecto la clasificación de un nivel alto o bajo de sedentarismo. Este enfoque nuevo e integrador parece ser una propuesta metodológica apropiada para clasificar el nivel de actividad física, con el objetivo de proporcionar a investigadores y profesionales de la salud una visión más amplia del comportamiento de la población frente a la actividad física.

## RESUMO

A inatividade física é um importante fator de risco contribuinte para a morbidade e a mortalidade em todo o mundo, embora o comportamento sedentário (CS), a atividade física de baixa intensidade (AFBI) e a redução da duração de sono estejam também associados a diversas doenças crônicas e estados fisiopatológicos potencialmente prejudiciais à saúde, qualquer que seja o nível de atividade física (AF). As metodologias atuais para avaliar e classificar o nível de AF de uma população têm limitações porque se baseiam sobretudo no tempo dispendido em AF de intensidade moderada a vigorosa (AFMV). O propósito deste artigo é examinar a literatura científica quanto às diversas combinações de padrões de sono, CS, AFBI e AFMV a fim de propor uma classificação de AF mais integrativa para crianças, adolescentes e adultos aparentemente saudáveis. A classificação de uso geral está dividida em quatro categorias que combinam AFMV com o nível de CS: i) fisicamente ativo ou fisicamente inativo (satisfaz ou não satisfaz os níveis recomendados de AFMV semanal) e ii) nível alto ou nível baixo de CS (segundo o tempo sedentário acumulado por dia). Existe consenso quanto à classificação de fisicamente ativo ou inativo, mas não quanto à classificação de nível alto ou baixo de CS. Esta nova abordagem integrativa é possivelmente uma proposição metodológica adequada para categorizar o nível de AF, pois oferece aos profissionais da saúde e pesquisadores uma concepção mais ampla dos comportamentos de atividade física na população.

In May 2013, the World Health Assembly endorsed the World Health Organization (WHO) global action plan for the prevention and control of noncommunicable diseases 2013-2020. That plan focuses on four shared behavioral risk factors: tobacco use, unhealthy diet, harmful use of alcohol, and physical inactivity (1). These modifiable risk factors make the largest contributions to morbidity and mortality in the world and can be considered some of the most relevant challenges for public health in the twenty-first century (1). In particular, the WHO has indicated that physical inactivity is strongly associated with three of the four main noncommunicable diseases: cardiovascular disease, cancer, and diabetes (1-3). In addition, physical inactivity is responsible for a substantial economic burden on health care systems (1).

Today, in highand middle-income countries, sedentary behavior (SB) is common among adults, who spend the majority of waking hours on such behaviors as watching TV, driving a car, and sitting while at work—all with relatively idle muscles (4). Sedentary behavior is defined by the Sedentary Behaviour Research Network as any waking behavior characterized by an energy expenditure ≤ 1.5 metabolic equivalents (METs), while the term “inactive” is used to describe individuals performing insufficient amounts of moderate-to-vigorous physical activity (MVPA) (5, 6).

Individuals who follow the physical activity (PA) recommendations developed by WHO (of at least 150 minutes of moderate-intensity (> 3METs) PA per week, or the equivalent) (1) are not exempt from the negative effects due to consistent SB (7). When combined with SB for most of the day, compliance with those recommended PA levels still presents an increased risk of several chronic conditions and mortality (4, 7).

Light-intensity PA (LIPA) has recently emerged as a strategy to replace sedentary activities (e.g., standing vs. sitting) since it might be more easily incorporated into daily life than is true for MVPA (8). LIPA consists of activities that are between > 1.5 and < 3.0 METs. Accumulating high levels of LIPA during the day has been linked to metabolic health benefits (8). Independent of age, MVPA, and other potential confounders, LIPA is inversely associated with all-cause mortality risk (9). Still, to date, LIPA has not been supported by international contemporary PA classification (1, 8, 10). It is important to note that replacing 30 minutes per day of SB with an equivalent time for LIPA or MVPA has been associated with an improvement in various health markers, such as triglycerides, insulin, the homeostatic model assessment (HOMA), waist circumference, and higher high-density lipoprotein cholesterol (11). These beneficial effects appear to be improved in an intensity-dependent manner (11, 12).

Sleep time has also been included as an important component in combined PA classifications, since shorter sleep duration is associated with adverse physical and mental health outcomes (10, 13). Daily time is finite; therefore, time spent in each of these three behaviors (sleep, SB, and PA) is codependent (12). MVPA is one of the most important and effective PA components related to the health status of an individual (4). However, ignoring other movement behaviors (such as LIPA, SB, and sleep time) when evaluating and classifying the PA level of the population appears to be a limited, although presently common, methodological approach (10).

## CURRENT PHYSICAL ACTIVITY CLASSIFICATION APPROACHES

Published in 2016, the *Canadian 24-Hour Movement Guidelines for Children and Youth* contained the first comprehensive PA recommendations in the world covering the three movement behavior components of PA, SB, and sleep (10). However, to date, in areas such as primary health care, preventive medicine, and public health, we do not have a PA classification approach that is supported by an integrative PA evaluation methodology.

Currently, PA levels are commonly measured in the general population (children, adults, and older persons) through so-called “objective methods” (e.g., accelerometer, pedometer), wearable devices to self-monitor physical activity (e.g., smartphone, smartwatches), or questionnaires (e.g., Global Physical Activity Questionnaire (GPAQ), International Physical Activity Questionnaires (IPAQ)). The common characteristic among the majority of these is a PA classification that focuses mainly on the amount of MVPA (while LIPA, SB, and sleep time are not included) and that generally uses dichotomized categories (compliance vs. noncompliance with the recommended minimum MVPA).

Developed by WHO as a tool for PA surveillance in countries, GPAQ has been used extensively in epidemiological studies around the world (14). It measures duration, frequency, and intensity of PA > 4 METs in three different PA domains. The first domain is “occupational” (at work), the second is “travel” (commuting activity), and the third is “leisure time” (recreational). The GPAQ also asks about SB (in terms of how much time a person usually spends sitting or reclining in a typical day), but that is not considered a GPAQ domain. When the three GPAQ PA levels (high, moderate, low) are assessed, SB is not considered. Sleep time is also not included in this questionnaire. Therefore, the most important PA questionnaire used by researchers and health professionals today appears to be incomplete. Further, the questionnaire does not take into account the accumulated body of evidence showing LIPA’s beneficial effect on health markers, and the detrimental effects of greater SB and of shorter sleep time (4, 8, 10, 13).

The performance of MVPA activities does not necessarily indicate a decrease in sedentary activities. Therefore, we must ask how to classify a person who meets the 150 minutes of MVPA per week (or 60 minutes per day in children) but is sitting for 12-14 hours each day, or who has a shorter or longer sleep time. The same type of question applies to an individual who does not meet the PA recommendation but spends large amounts of time doing LIPA, thus reducing his or her SB time.

The use of wearable devices to self-monitor PA has increased exponentially over the past decade, thereby offering individuals the opportunity to track and meet PA recommendations and thus prevent chronic disease. However, current PA guidelines were not formulated with such technology in mind. Thomson et al. (15&oint out that since data from these devices overestimate the MVPA of individuals, the recommendation should be for around 1 000 minutes per week, which represents about 15% of waking time. Conversely, two individuals could accumulate the same energy expenditure during a day, but through dissimilar patterns of behavior, due to differences in the amount of time spent in sleep, SB, LIPA, and MVPA (16). These research studies therefore emphasize the importance of the method used to quantify PA in a population, as well as how PA is accumulated during the day. Both these factors have strong implications for the future of PA classification.

## THE TRANSITION TOWARD AN INTEGRATED CLASSIFICATION OF PHYSICAL ACTIVITY LEVELS

From analyzing the scientific literature, we can see that some studies have begun to explore different combinations of patterns among sleep, SB, LIPA, and MVPA in order to categorize children, adolescents, and adults in a more integrative way (17-21). Most of these strategies employ “objective methods,” mainly accelerometry. Overall, a consensus seems to have emerged in scientific literature with respect to classifying a person as “physically active” or “physically inactive” through the WHO PA recommendation (1, 14). However, there is no general agreement regarding the use of a specific cutoff point to determine a high SB or low SB level. Use of sleep time in this kind of integrative PA classification appears limited (19).

## COMPUTING AN INTEGRATIVE PHYSICAL ACTIVITY CLASSIFICATION

The majority of studies in the current literature on integrative PA classification consider four categories (18, 21, 22). The first two categories arise from level of MVPA (or equivalent, e.g., ≥ 600 METs per week), and the two other categories come from the interaction between SB and LIPA time. For example, considering current population-based data, Loprinzi et al. (22) classified a sample of 5 580 adults over 20 years old from NHANES 2003-2006 into four groups. Using accelerometry, those researchers found that of the sample who engaged in ≥ 150 min/week of MVPA, 16.2% had LIPA > SB and 27.7% had LIPA < SB. They also found that of the sample who engaged in < 150 min/week of MVPA, 8.7% had LIPA > SB and 47.2% had LIPA < SB (22).

### First subclassification: physically active or inactive

Currently, we can use accelerometers, questionnaires, pedometers, and a question about muscle strengthening to establish if a person is meeting one of the several PA recommendations proposed by different international organizations (1, 23-26) (Table 1). Depending on the results, the person will be categorized as either “physically active” or as “physically inactive.”

### Second subclassification: high or low sedentary behavior

The methods used for measurement of SB and the corresponding analytical methodology appear to be the most important factors that hinder a consensus regarding the use of a more integrative approach to PA classification. Both accelerometers and questionnaires can determine the amount of SB time, allowing us to mainly compute two categories (high SB or low SB). It is possible to find other categories in the scientific literature, including the 50th percentile (median), tertiles, and quartiles (17, 18, 20, 21). However, tertiles and quartiles are usually eventually dichotomized into high SB or low SB (Table 1).

Unlike the questionnaires, accelerometers also allow us to determine LIPA time, and some studies have used a SB ÷ LIPA ratio (18) or LIPA > SB (17, 22) to compute a continuous variable that is also capable of being split. Most of these variables use a percentage of LIPA and SB time to adjust data to accelerometer wear time in their analysis (17).

## IMPLICATIONS OF THIS NEW PHYSICAL ACTIVITY CLASSIFICATION

**FIGURE 1.**
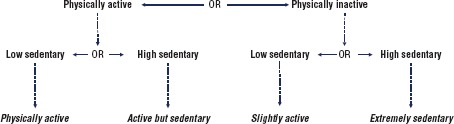
Integrated classification of physical activity levels

***Source:*** Prepared by the author.

PA is a multidimensional construct composed of diverse behaviors that interact within a finite period of time. Therefore, any change to even one behavior could influence health status (11, 12, 15, 16). In the view of this author, having a common and integrative PA classification methodology in public health (Figure 1) will allow health professionals and researchers to generate prescriptions, treatments, and interventions that are more progressive and tailored to the individual (e.g., reallocating SB time to LIPA in “extremely sedentary” subjects, or mainly increasing MVPA in “active but sedentary” subjects). This would also facilitate comparison among PA categories in distinct populations (e.g. children, adults, the elderly). Further, it is of substantial interest in preventive medicine to promote strategies to avert and control such chronic diseases and conditions as heart disease,stroke, cancer, diabetes, and obesity (2, 23). In this sense, PA intervention is a low-cost, accessible, safe strategy to improve the general health status of children, adolescents, adults, and elderly persons.

**TABLE 1.** Methodology to compute an integrative physical activity classification

Subclassification and methods for its determination	Specifications for each measurement method (ref(s).)
First subclassification: physically active or physically inactive Accelerometers and questionnaires	≥ 60 minutes MVPA^a^/day = physically active (children) (1, 23, 24) ≥ 150 minutes MVPA/week = physically active (adults) (1, 23, 24) ≥ 150 minutes MPA^b^/week = physically active (older adults) (1, 23, 24) ≥ 600 METs^c^/minute/week = physically active (adults) (24)
Pedometer	≥ 11 700 steps per day (children) (26) ≥ 10 000 steps per day (adults) (26) ≥ 8 000 steps per day (older adults) (26)
Muscle strengthening	≥ 8 sessions within the past 30 days (adults) (25)
Second subclassification: low sedentary behavior (SB) or high SB Accelerometers and questionnaires	SB time Median: below the 50th percentile = low SB (21) Tertiles 1 and 2 = high SB; Tertile 3 = low SB (20) Quartiles 1, 2, 3 = high SB; Quartile 4 = low SB (18)
Only accelerometers	SB ÷ LIPA^d^ (18) LIPA > SB time = low SB (17)

***Source:*** Prepared by the author.

a MVPA = moderate-to-vigorous physical activity.

b MPA = moderate physical activity.

c METs = metabolic equivalents.

d LIPA = light-intensity physical activity.

Figure 1 shows the most common PA classification used by researchers today. This is composed of four categories that combine a dichotomized subclassification for MVPA and for SB. Sleep time is not included. That is because sleep time presents certain inconsistencies in the scientific literature to date (13, 27), so to integrate it in this approach would be premature. In this regard, there is no evidence on whether meeting the recommendations for specific behaviors (such as sleep vs. PA vs. SB) would present similar associations with various health indicators (19), which in turn reinforces the methodology presented in Figure 1. In this manner, this methodology to classify PA for the general population allows future evaluations of the main behaviors that involve a large portion of energy expenditure and time use during a day.

## LIMITATIONS AND STRENGTHS OF THIS APPROACH

Among the limitations of this approach are that: a) there is a lack of studies using an integrative PA classification, while even fewer utilize the important component of sleep time; b) cutoff values for SB, LIPA, and MVPA depend largely on the method used to quantify PA (accelerometers, wearables devices, or questionnaires), which makes it difficult to achieve a common strategy; c) a systematic review is needed in order to reinforce this methodological approach; d) the terminology presented in Figure 1 is a personal proposal that considers the wide range of possibilities published in the scientific literature to date; it is not derived from formative work, such as a focus group; and e) LIPA will need to be included in future PA questionnaires in order to apply this kind of integrative classification to large populations.

Among the strengths of this approach are that: a) to date, various approaches have been published to recommend PA in a more integrative way but not to classify populations by considering all health-linked movement behaviors in a 24-hour period; b) this article proposes a type of guide for researchers and health professionals to categorize individuals by using a more integrative approach that employs one method or a combination of methods; and c) it appears to be the first article that considers the scientific evidence published to date and proposes a classification of this type.

## CONCLUSIONS

In order to help prevent noncommunicable diseases and reduce mortality, it is essential to consider the importance of sedentary behavior, low-intensity physical activity, moderate-to-vigorous physical activity, and even sleep duration. As part of this effort, it is necessary to include those categories in future global PA questionnaires. Moving toward that new and more integrative methodological approach will help deliver a broader vision of PA behaviors in the world population.

### Disclaimer

The author holds sole responsibility for the views expressed in the manuscript, which may not necessarily reflect the opinion or policy of the *RPSP/PAJPH* or PAHO.
